# A rare case of narrow QRS complex tachycardia

**DOI:** 10.1007/s12471-014-0616-y

**Published:** 2014-11-11

**Authors:** L. E. Swart, Y. S. Tuininga

**Affiliations:** Department of Cardiology, Deventer Hospital, Nico Bolkesteinlaan 75, 7416 SE Deventer, the Netherlands

**Fig. 1:**
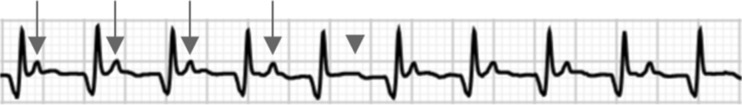
Retrograde Wenckebach behavior

The first ECG shows a regular narrow QRS complex tachycardia of approximately 160 beats/min. Clear P waves can be distinguished closely behind each QRS complex. These same P waves appear to be negatively deflected in the inferior leads, which could fit an atrioventricular (nodal) reentrant tachycardia (AVNRT) with retrograde P waves. In the next ECG, the R-P time appears to increase with every beat (arrows in Fig. 1), until a QRS complex (the fifth) is no longer followed by a P wave (triangle in Fig. 1). This is retrograde Wenckebach behaviour, in which the retrograde P wave appears to be an innocent bystander.

**Fig. 2:**
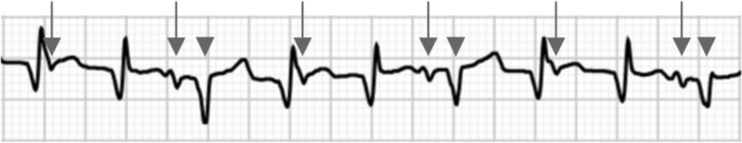
AV dissociation with fusion or capture beats

But it is not until the third ECG that we notice there is no longer any association between the QRS complexes and P waves. In this ECG, the morphology of the P wave has also changed, indicating a normal sinus rhythm (arrows in Fig. 2) with AV dissociation. In the rhythm strip at the bottom, we also notice a different QRS complex every third beat for the first 20 beats. This different QRS complex appears to be more narrow (approximately 80–90 ms) and is each time preceded by a sinus P wave, suggesting these might be fusion or capture beats (triangles in Fig. 2).

These two findings combined lead to the conclusion that this rhythm can only be a ventricular tachycardia, most likely originating near or from the His-Purkinje system, resulting in a narrow QRS complex. Sinus rhythm was restored after intravenous administration of 300 mg of amiodarone.

A narrow QRS ventricular tachycardia (VT) is not unusual, and has previously been described in patients with ischaemic heart disease [1, 2]. It has been reported in cases of idiopathic VT but mostly originates from structural heart disease in the His-Purkinje area, and has therefore been called fascicular tachycardia [3, 4]. In a selected group of 106 patients with VT, Hayes et al. reported five patients who had a QRS duration of less than 110 ms [5]. Four of these five patients underwent electrophysiological mapping during surgery and the focus of their VT was mostly mapped to the ventricular septum, close to the proximal conduction system, as with the location of the infarction in our patient.

In this case, narrow QRS VT mimicked a supraventricular tachycardia. The various presentations of the P waves in the three consecutive ECGs (retrograde 1:1 conduction, retrograde Wenckebach and AV dissociation) made this a compelling case.
